# The Sick Chamber at Night

**Published:** 1872-01

**Authors:** 


### THE SICK CHAMBER AT NIGHT.

The glare of a bright light at night tends to keep an invalid from sleeping; and yet, in many cases, it is not desirable to be in total darkness. If a burning candle has common salt put on the melted part of the candle, until it reaches the black part of the wick, it will not only cause the candle to burn very slow, but make it give a dim, mild, and mellow light, proving very agreeable to the invalid.

Another expedient in this connection: a room can be very well ventilated, in the hotter nights, by lighting a candle and placing it on the hearth in the fire-place; this causes a draught upwards, which is promoted by the fresh air coming in at an open window or door. In the winter, a fire should be always burning in the grate or fire-place, more necessary at night than in the daytime; it not only keeps the air of the room pure and good, but it prevents the room getting too cool, thus endangering pneumonia or lung fever, in proportion as the invalid is debilitated.

